# Two-Step Exfoliation of WS_2_ for NO_2_, H_2_ and Humidity Sensing Applications

**DOI:** 10.3390/nano9101363

**Published:** 2019-09-24

**Authors:** Valentina Paolucci, Seyed Mahmoud Emamjomeh, Michele Nardone, Luca Ottaviano, Carlo Cantalini

**Affiliations:** 1 Department of Industrial and Information Engineering and Economics, Via G. Gronchi 18, University of L’Aquila, I-67100 L’Aquila, Italy; seyedmahmoud.emamjomeh@graduate.univaq.it (S.M.E.); carlo.cantalini@univaq.it (C.C.); 2 Department of Physical and Chemical Sciences, Via Vetoio 10, University of L’Aquila, I-67100 L’Aquila, Italy; michele.nardone@univaq.it (M.N.); luca.ottaviano@aquila.infn.it (L.O.); 3 CNR-SPIN Uos L’Aquila, Via Vetoio 10, I-67100 L’Aquila, Italy

**Keywords:** 2D-materials, WS_2_, exfoliation, gas sensors, NO_2_, H_2_, cross sensitivity

## Abstract

WS_2_ exfoliated by a combined ball milling and sonication technique to produce few-layer WS_2_ is characterized and assembled as chemo-resistive NO_2_, H_2_ and humidity sensors. Microstructural analyses reveal flakes with average dimensions of 110 nm, “aspect ratio” of lateral dimension to the thickness of 27. Due to spontaneous oxidation of exfoliated WS_2_ to amorphous WO_3_, films have been pre-annealed at 180 °C to stabilize WO_3_ content at ≈58%, as determined by X-ray Photoelectron Spectroscopy (XPS), Raman and grazing incidence X-ray Diffraction (XRD) techniques. Microstructural analysis repeated after one-year conditioning highlighted that amorphous WO_3_ concentration is stable, attesting the validity of the pre-annealing procedure. WS_2_ films were NO_2_, H_2_ and humidity tested at 150 °C operating Temperature (OT), exhibiting experimental detection limits of 200 ppb and 5 ppm to NO_2_ and H_2_ in dry air, respectively. Long-term stability of the electrical response recorded over one year of sustained conditions at 150 °C OT and different gases demonstrated good reproducibility of the electrical signal. The role played by WO_3_ and WS_2_ upon gas response has been addressed and a likely reaction gas-mechanism presented. Controlling the microstructure and surface oxidation of exfoliated Transition Metal Dichalcogenides (TMDs) represents a stepping-stone to assess the reproducibility and long-term response of TMDs monolayers in gas sensing applications.

## 1. Introduction

In recent years, layered materials such as two-dimensional (2D) transition metal dichalcogenides (TMDs) have attracted a high level of interest due to their features, which make them appealing for potential applications in gas sensing [1,2], photo-electro-catalytic hydrogen evolution [3,4] optical and electronic devices [5,6] and energy storage [7,8].

Few-layer 2D TMDs can be produced through different methodologies like mechanical and liquid phase exfoliation of bulk crystals, classified as top-down routes, or via direct bottom-up routes like chemical vapor deposition [9,10]. High-yield liquid exfoliation methods comprising ion intercalation [11,12] and ultrasonic cleavage [13,14] have also been widely employed to exfoliate bulk-layered materials. Besides liquid phase exfoliation, low energy ball milling as a newly explored high-yield mechanical exfoliation method has been utilized for scalable production of mono and few-layer graphene [15,16] and TMDs nano-sheets [17,18]. More recently, enhanced mixed methods comprising assisted grinding and sonication have been shown to produce higher concentrations of TMDs nano-sheets and a reduced amount of defects [19].

Regarding gas sensing applications, 2D single layer MoS_2_ Field Effect Transistor [20,21] Pd-doped WS_2_ films [22] have been shown to be alternative substitutes for traditional metal oxides sensors. Moreover, considering that the possibility to find practical applications of those materials is generally dependent on the reproducibility of the preparation with respect to both microstructure (i.e., number of layers, lateral size, surface area, etc.) and chemical composition (i.e., defects concentration and surface oxidation), the need to find a practical, high-reproducible, and easy way to exfoliate TMDs is always under investigation. We already presented the fabrication of chemo-resistive thin films gas sensor, by drop casting suspensions of few flakes graphene oxide [23], phosphorene [24] and more recently TMDs utilizing both liquid-exfoliated MoS_2_ [25] and commercially exfoliated suspensions of WS_2_ [26]. The aim of this work is to apply a low energy ball milling and sonication method to achieve a reproducible and high-yield exfoliation methodology and to test the gas sensing performances of the obtained material. Starting from commercial WS_2_ powders, we have firstly performed the exfoliation process based on the grinding and sonication method and investigated the morphology of few flakes WS_2_. Secondly, we have determined that with exposing the material to mild air annealing at 180 °C, a controlled partial oxidation of WS_2_ flakes to amorphous WO_3_ is achievable. Lastly, we investigated the sensing responses to NO_2_, H_2_ and humidity of drop casted exfoliated WS_2_ chemo-resistive thin films, discussing the likely gas-response mechanism.

## 2. Materials and Methods

*WS_2_ exfoliation*: According to the flow sheet shown in Supplementary Figure S1, 2 g of WS_2_ commercial powder (Sigma–Aldrich 243639-50G, St. Louis, MO, USA) were dispersed in 4 mL Acetonitrile (ACN—VWR 83639.320, Radnor, PA, USA) with 30 g Zirconium Oxide balls (D = 3 mm), and ball milled in a planetary milling machine (Fritsch—Planetary Micro Mill Pulverisette 7, Idar-Oberstein, Germany) at 400 rpm, for 2 h in ambient air.

To evaporate ACN residuals after milling, the collected slurry was left overnight at 23 ± 2 °C temperature and 40% ± 3% Relative Humidity (RH) (ATP DT-625 High Accuracy Thermo-hygrometer, Ashby-de-la-Zouch, UK). After ACN evaporation, 0.05 g of the dried powder was dispersed in 100 mL of pure ethanol (99.94% VWR 20821.330, Radnor, PA, USA) and probe sonicated (Sonics VC 505 ultrasonic processor, Newtown, CT, USA) at 250 W for 90 min in a thermostat bath to prevent temperature rise (T 25 °C). Finally, the solution was centrifuged at 2500 rpm for 40 min in a refrigerated (20 ± 2 °C) micro-centrifuge (Eppendorf 5417R, Hamburg, Germany) and the supernatant collected.

*Microstructural and chemical characterization*: Air tapping mode Atomic Force Microscopy (AFM) was performed with a Veeco Digital D5000 system. Using silicon tips with spring constant of 3 N∙m^−1^ and resonance frequencies between 51 and 94 kHz. Samples for AFM investigations were prepared via spinning (at 2000 rpm for 30 s) 10 μL of centrifuged WS_2_/Ethanol solution on a Si_3_N_4_ substrate. The substrates have been previously cleaned in a piranha base solution (3:1:3 mixture of ammonium hydroxide NH_4_OH with hydrogen peroxide and milli-Q water) to enhance their wettability.

Exfoliated flakes were investigated using High Resolution Transmission Electron Microscopy HRTEM—JEOL 2100 Field Emission electron microscope (Tokyo, Japan) operated at 200 kV. Samples prepared by drop casting the WS_2_/Eth solution on Si_3_N_4_ substrate were investigated by X-Ray Photoelectron Spectroscopy (XPS) using a PHI 1257 spectrometer (Perkin Elmer, Norwalk, CT, USA) equipped with a monochromatic Al Kα source (hν = 1486.6 eV) with a pass energy of 11.75 eV (93.9 eV survey), corresponding to an overall experimental resolution of 0.25 eV. Raman spectra were acquired using a Micro Raman Spectrometer (μRS) (LABRAM spectrometer, λ = 633 nm, 1 μm spatial resolution, and ≈2 cm^−1^ spectral resolution, Horiba-Jobin Yvon, Kyoto, Japan) equipped with a confocal optical microscope (100 × MPLAN objective with 0.9 numerical aperture and 0.15 mm work distance). 10 μL of the WS_2_/Eth solution was deposited on a 270 nm SiO_2_ substrate.

*Gas sensing measurements*: Electrical properties were determined by a volt-amperometric technique (AGILENT 34970A), as reported in Supplementary Figure S2, utilizing WS_2_ thin films prepared by multiple drop casting and air annealing at 180 °C for 1 h the centrifuged WS_2_/Eth suspension on Si_3_N_4_ substrates provided with 30 μm-spaced Pt interdigitated electrodes on the front side and a Pt resistor acting as a heater on the back side. Different gas concentrations in the range 1 ppm–250 ppm H_2_ and 40 ppb–5 ppm NO_2_ were obtained by mixing certified H_2_, and NO_2_ mixtures with dry air carrier, by means of an MKS147 multi gas mass controller. Different relative humidity (RH) air streams in the 10–80% RH range were obtained by mixing dry with saturated water-vapor air. The following definitions apply to discuss the gas response properties: base line resistance (BLR): the resistance in dry air at equilibrium before gas exposure, relative response (RR): the ratio (R_A_/R_G_) where R_A_ represents the resistance in air and R_G_ the one in gas at equilibrium for a given gas concentration, and sensor sensitivity (S): is the slope of the calibration curve in the sensitivity plot.

## 3. Results and Discussion

### 3.1. Microstructural Properties of Exfoliated WS_2_

The microstructure of exfoliated WS_2_ obtained by the combined ball milling and sonication process is characterized. In our case, acetonitrile (ACN) as the milling solvent, with surface tension of 29.5 mJ m^−2^ [27], has been selected as a trade-off between surface tension and moderate boiling point, enabling complete removal of the solvent at room temperature after grinding.

**Figure 1:**
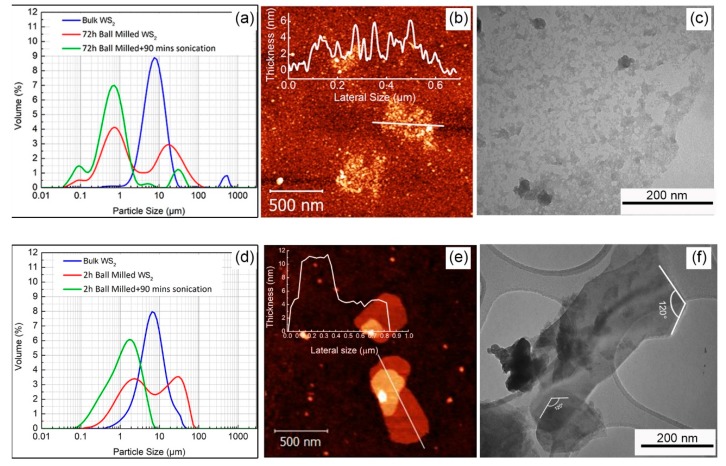
Comparison of the effect of long-time ball milling on WS_2_ exfoliation: (**a**) Comparison of the particle size distribution of the starting WS_2_ commercial powder (blue), 72 h ball milled (red) and 72 h ball milled and 90 min sonicated (green), (**b**) AFM picture of 72 h ball milled and 90 min sonicated and associated thickness profile along the white line, (**c**) TEM picture of the 72 h ball milled and 90 min sonicated WS_2_, (**d**) Comparison of the particle size distribution in case of 2 h ball milling, (**e**) AFM picture of 2 h ball milled and 90 min sonicated and associated thickness profile, (**f**) TEM picture of the flakes obtained by 2 h ball milling and 90 min sonication of WS_2_.

Regarding the influence of the grinding time, particles’ size distribution of the starting WS_2_ powder (blue plot), determined by Dynamic Light Scattering (DLS) technique (see experimental section) and shown in Figure 1a, downshifts towards smaller average sizes after 72 h grinding (red plot) and slightly further after 90 min sonication (green plot). The particle size distribution of the WS_2_ starting powder displays an average particle size of ≈ 8 μm (blue plot) whereas the 72 h ball milled shows a bimodal distribution (red plot), with larger aggregates centered at ≈ 20 μm and smaller ones at ≈ 0.7 μm. After 90 min sonication, the bimodal distribution of the grinded powder disappears (green plot) and the average particle dimension places at ≈ 0.6 μm.

It may be concluded that grinding has an effective influence to reduce the particle size, while sonication, beside its effectiveness to suppress the bimodal distribution (presumably by separating agglomerated WS_2_ particles), shows only minor effects to further decrease the particle size of the grinded powder, confirming the dominant role of the milling step. Figure 1b shows the AFM image of the 72 h ball milled and 90 min sonicated WS_2_ sample. The inset of this image depicts a rough thickness profile along the selected line, with an average height from the substrate of 2 nm. Low and high magnification TEM images illustrated in Figure 1c,d, show that long term ball milling for 72 h results in a fragmented structure, which was eventually revealed to be amorphous by fast Fourier electron diffraction measurements. These features can be explained considering the two main forces induced by ball milling. The primary force is the shear force provided by rolling of balls on the surface of layers, which causes the removal and the exfoliation of surface layers. The secondary force is the vertical impact from the balls which combined with longer grinding times can fragment the larger exfoliated sheets into smaller ones, eventually collapsing of the crystal structure [15,28].

With the aim to minimize the fragmentation effect, we have reduced the ball milling duration time from 72 to 2 h, maintaining the sonication time at 90 min. Decreasing the milling time to 2 h, the flake’s fragmentation sharply decreases, enforcing the formation of well-defined terraced structures comprising stacked WS_2_ flakes, as shown in Figure 1e,f.

**Figure 2:**
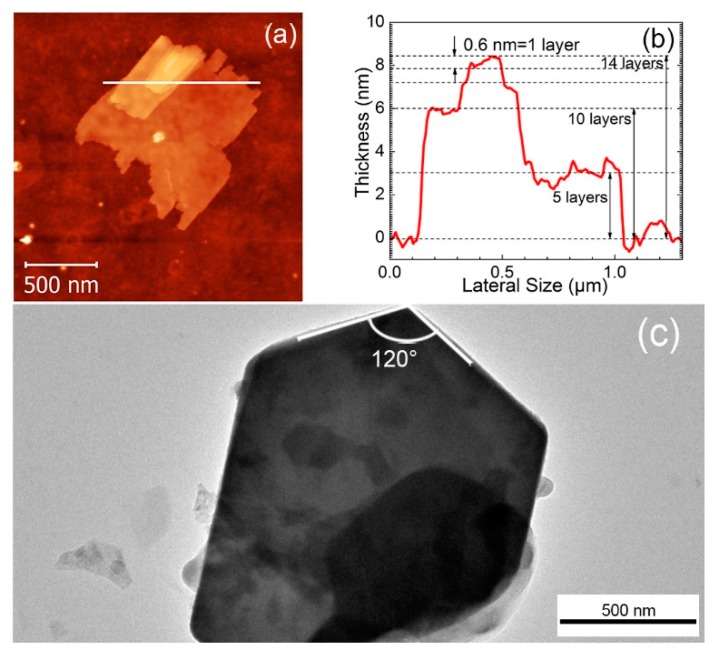
(**a**) AFM picture of the 2 h ball milled and 90 min sonicated WS_2_, (**b**) thickness profile of the stacked flake along the white line of Figure (**a**), (**c**) low-resolution TEM picture of an exfoliated flake.

Figure 2 shows the main microstructural features of the 2 h ball milled and 90 min sonicated WS_2_ powders. The AFM image shown in Figure 2a depicts the formation of a well-shaped 2D-flake with a large flat surface of 1 μm length. The corresponding thickness profile drawn in Figure 2b highlights a clear formation of a stacked structure comprising a 3 nm thick basal plane, 6 nm thick secondary plane and a third one at the top. Considering that the slight step on top of the profile, 0.6 nm high, corresponds to 1 layer thickness WS_2_ [29], it is shown that the first step is made of 5 layers and the second one of 10 layers, respectively. The 2D character of the actual stacked structure shown in Figure 2b, as defined by the “aspect ratio” (i.e., the ratio of lateral dimension to the thickness), is high, with an associated value of 250, attesting to the successful optimization of the grinding time for exfoliation. The low-resolution TEM image depicted in Figure 2c also illustrates a well-shaped exfoliated flake with edge angles of 120°, confirming the preservation of the typical crystalline WS_2_ hexagonal geometry.

**Figure 3:**
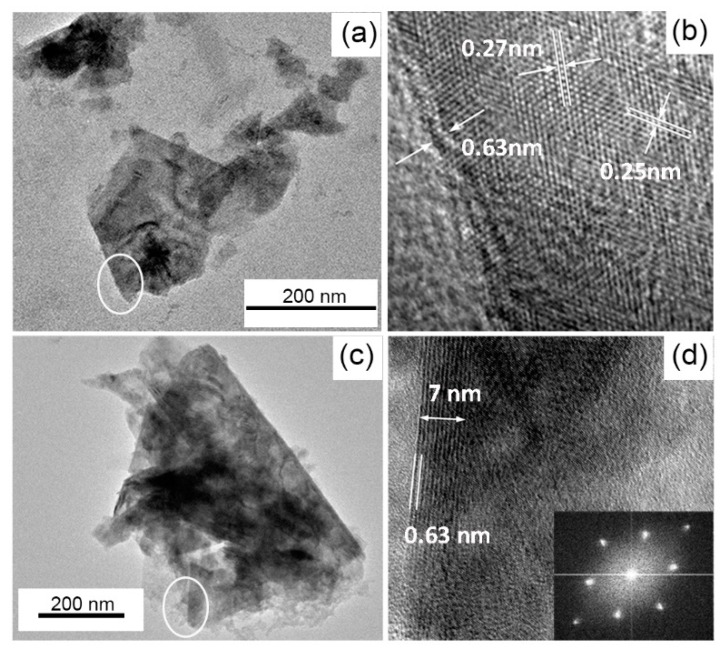
(**a**,**c**) TEM of 2 h ball milled and 90 min sonicated WS_2_, (**b**) HRTEM corresponding to the circled area shown in figure (**a**) with highlighted the interlayer distance (0.63 nm) and lattice spacing (0.27 nm and 0.25 nm), corresponding to (100) and (101) planes of WS_2_ respectively, (**d**) HRTEM of the edge of the flake corresponding to the circled area shown in figure (**c**) with highlighted the 7 nm thick edge corresponding to 11 layers. The inset shows the Selected Area Electron Diffraction (SAED) of the flake.

Figure 3 shows HRTEM images of both edges and surfaces of the flakes. Figure 3b (the magnification of Figure 3a), exhibits two layers with associated interlayer distances of ≈ 0.63 nm which is in good agreement with the AFM thickness measurements illustrated in Figure 2b. This interlayer displacement could also be observed at the flake’s edges depicted in Figure 3d, where almost 11 layers can be clearly counted on the 7 nm thick edge. The atoms arrangement displayed in Figure 3b exhibits the hexagonal atomic structure, with lattice spacing of 0.27 nm and 0.25 nm, that are characteristics of (100) and (101) crystal planes of 2H-WS_2_ flakes, respectively [30,31]. Moreover, the Fast Fourier Transforms (FFTs) shown as the inset of Figure 3d, further confirms the hexagonal crystalline structure of the flake.

**Figure 4:**
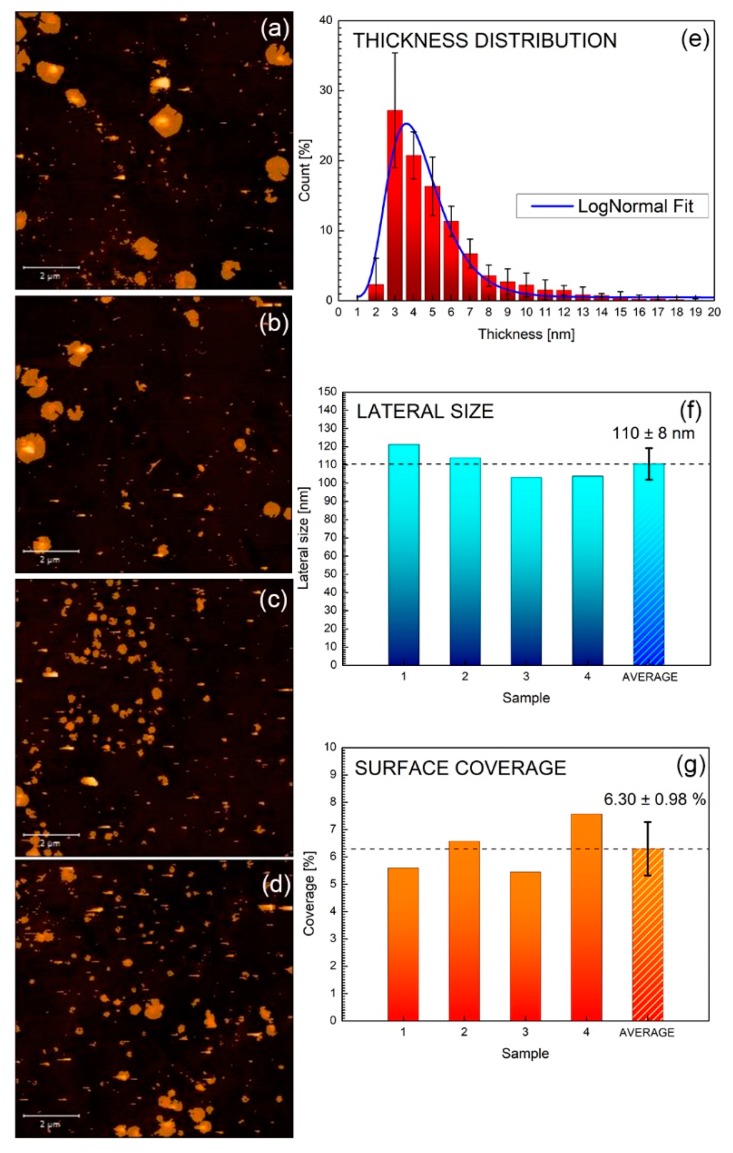
(**a**–**d**) AFM images of WS_2_ exfoliated corresponding to four different samples prepared under the same conditions (i.e., 2 h ball milling and 90 min sonication). Statistical analysis corresponding to thickness distribution (**e**), Lateral dimensions (**f**) and surface area coverage (**g**).

To give a statistical insight of the reproducibility of the preparation, four different suspensions were prepared after 2 h milling and 90 min sonication and the corresponding centrifuged suspensions collected, and spin coated on Si_3_N_4_ substrates (see experimental section). Figure 4a–d shows the AFM images of each prepared sample covering an area of 10 × 10 μm^2^ and corresponding to a total population of ≈ 220 flakes. Overall, flakes’ thickness follows a log-normal distribution as shown in Figure 4e, indicating that almost 30% of the flakes are ≤3.0 nm thick (i.e., ≈ 5 layers) and that about 75% are ≤6 nm (i.e., ≈ 10 layers). Moreover, as displayed in Figure 4f, average flake lateral dimensions are approximately ≈ 110 nm, yielding a surface coverage of ≈ 6%, as shown in Figure 4g. The overall calculated “aspect ratio” is 27.5, which is comparable to the ones previously reported for MoS_2_ and WS_2_, given the same preparation methodology [27,32]. The reduced standard deviations shown in Figure 4f,g attest to the high reproducibility of the exfoliation process.

### 3.2. Chemical Composition of the Exfoliated WS_2_

Chemical issues related to both the evolution of point defects and oxidation phenomena of TMDs highlight important challenges associated with the practical utilization of TMDs monolayers in electronic and optoelectronic devices. Sulphur vacancy is one of the most typical point defects in 2D MoS_2_ and WS_2_ monolayers [33], eventually leading to active sites for gas adsorption. On the other hand, spontaneous oxidation of metal sulphides into their metal oxide counterparts [34], may result in poor reproducibility of the gas-sensing response over the long-term, as discussed in our previous publication [26].

**Figure 5:**
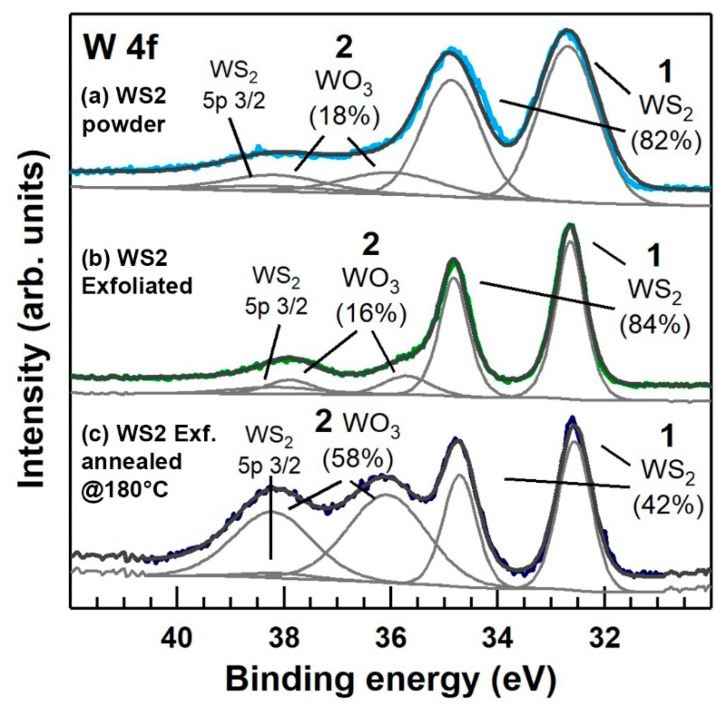
X-Ray Photoemission Spectroscopy (XPS) spectra of W 4f core level acquired respectively on (**a**) pristine WS_2_ commercial powder (WS_2_ PWD), (**b**) Exfoliated WS_2_ by ball milling and sonication at 25 °C, (**c**) WS_2_ exfoliated and post-annealed at 180 °C. All the components and their relative atomic percentages are labelled in the figure.

Figure 5, panel (a), reports the W 4f core level XPS spectra of the pristine commercial WS_2_ powder (PWD). The two doublets corresponding to 4f_7/2_ peaks are assigned, according to the literature, to WS_2_ and WO_3_, respectively [35,36]. It turns out that the pristine powder is already oxidized, with a WO_3_ content of ≈ 18%. This phenomenon, is not surprising considering that spontaneous oxidation of MoS_2_ at room temperature after 6–12 months has been already reported in the literature, demonstrating the occurrence of ageing phenomena of MoS_2_ [25] and WS_2_ monolayers in ambient air [26]. Figure 5b shows that after exfoliation, the WO_3_ content decreases to ≈ 16%. This could be explained considering that by grinding and sonicating WS_2_ powders, newly not-yet-oxidized surfaces are formed, resulting in a smaller content of WO_3_, as evidenced by XPS measurements. Furthermore, as discussed in the next paragraph, given the optimum operating temperature for gas sensing at 150 °C, exfoliated WS_2_ suspensions, were therefore drop-casted and pre-annealed at 180 °C for 1 h in air to stabilize the oxidation levels. As shown in Figure 5c, after annealing, the WO_3_ content increased to ≈ 58%.

Regarding sulphur vacancies formation, considering 1% the detection limit of the XPS measurement, we found no clear evidence of defects of sulfur vacancies-related components (typically at binding energies of ≈ 36.1 in the W 4f core level XPS spectra in Figure 5). The analysis of the S 2p core level XPS spectra, reported in Supplementary Figure S3, is in line with the analysis reported for the W 4f.

**Figure 6:**
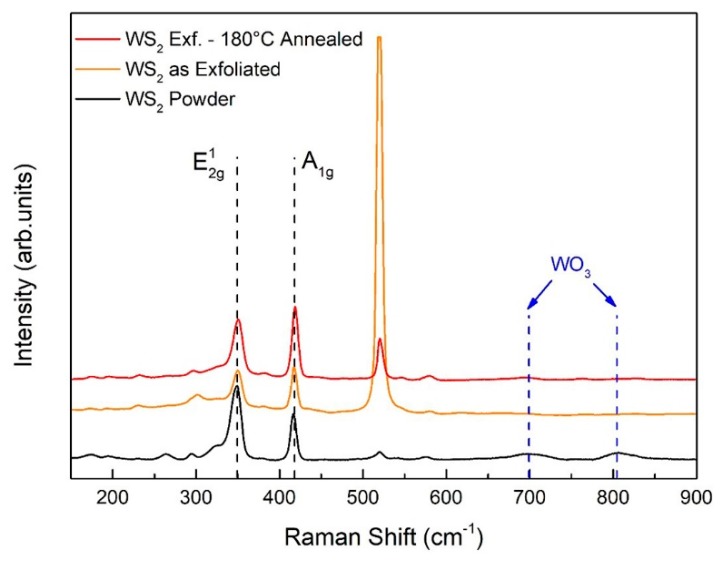
Raman spectra of WS_2_ bulk powder, WS_2_ as-exfoliated and WS_2_ flakes post-annealed at 180 °C.

Figure 6 displays the Raman spectra of bulk powder, exfoliated flakes and 180 °C annealed film. Peaks located at 350 and 419 cm^−1^ refer to crystalline WS_2_. The peak at 520 cm^−1^ corresponds to the substrate (i.e., crystalline SiO_2_). Raman spectra reveal that neither crystalline nor amorphous WO_3_ are formed. According to the literature [37], the displacement of crystalline WO_3_ is excluded considering that no peaks corresponding to the dashed lines at 719 and 807 cm^−1^ are observed. No signals of amorphous WO_3_ were shown, as attested by the absence of a broad peak between 600 and 900 cm^−1^, attributed to the W-O stretching vibration of amorphous WO_3_. The lack from the Raman spectra of any WO_3_ signal, as opposed to XPS, can be explained considering that Raman spectroscopy penetrates more deeply inside the material, suggesting that the overall amount of WO_3_ throughout the whole flakes is negligible.

In order to have a better understanding where the as-formed WO_3_ is located, firstly we have to consider that both XPS and Raman techniques give information on the chemical bonding of the elements, secondly that XPS information comes from photoelectrons escaping maximum up to 10 nm below the material surface, lastly that Raman spectroscopy, compared to XPS, is a “bulk” technique (given the negligible attenuation of visible light at the length scale of microns). It turns out that the WS_2_/WO_3_ percentage content measured by XPS and shown in Figure 4 (i.e., ≈ 58%), represents the average chemical compositions of a portion of the material confined within at last 10 nm from the material surface. This region, for simplicity, can be referred as a “surface layer”, which represents the reacting surface to interfering gases. Regarding the crystallographic nature of the “surface layer”, grazing incidence XRD diffraction carried on exfoliated and 200 °C annealed WS_2_ flakes (shown in Supplementary Figure S4) revealed the occurrence of peaks belonging only to WS_2_, thus excluding the formation of any crystalline WO_3_ in the 180 °C annealed film. It may be concluded that the “surface layer” is a composite structure comprising amorphous WO_3_ and pristine crystalline WS_2_. These experimental results are in line with that previously discussed in the literature [38], that the oxidation of bulk TMDs provides two parallel steps. Oxygen atoms rapidly exchange with surface sulphur forming an amorphous oxide layer, whilst WS_2_ interlayer channels provide a path for inward-oxygen and backward-sulphur diffusion, resulting in the formation of amorphous WO_3_, which propagates over time inside the TMD flakes.

### 3.3. Gas Sensing Response

It has been reported that TMDs gas sensors operating at room temperature have shown remarkable limitations, largely related to irreversible desorption of the gas molecules, displaying incomplete recovery of the baseline at 25 °C [39,40]. The selection of the best operating temperature (OT), which it corresponds the complete base line recovery, within reasonable response times, and acceptable gas relative responses (RR), is limited in TMDs by the intensifying of the oxidation processes with increasing the OT, as previously discussed. Baseline recovery and response times depend on the adsorption-desorption kinetic of gases with the sensor surface, which eventually improves with increasing the OT. RR is mostly related to microstructure (i.e., surface area, grain size), concentration of surface defects (i.e., oxygen or sulphur vacancies) and structure of the reacting surface (crystalline or amorphous). To find the best OT exfoliated WS_2_ drop casted on Si_3_N_4_, substrates were previously air annealed at 180 °C to stabilize the WO_3_ content up to ≈ 58%, as shown in Figure 5c. Afterwards, different OT in the 50–150 °C temperature range where tested, resulting in 150 °C as the best OT, as shown in Supplementary Figure S5.

**Figure 7:**
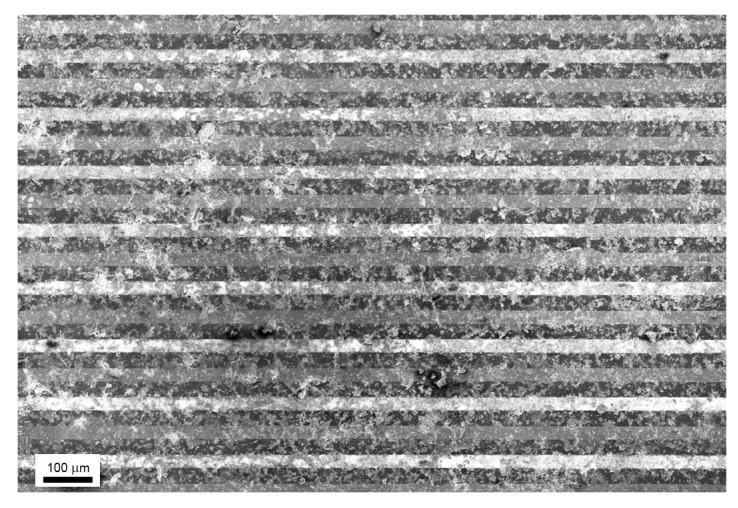
SEM image of sensor obtained by drop casting exfoliated WS_2_ and annealing at 180 °C on Si_3_N_4_ substrate provided with Pt finger-type electrodes (30 microns apart).

The Scanning Electron Microscopy (SEM) picture of the sensing device shown in Figure 7 highlights a homogeneous distribution of annealed flakes, enabling current percolation paths between adjacent flakes, covering an area of 1.4 × 0.6 mm^2^ over 30 μm spaced Pt interdigitated electrodes.

**Figure 8:**
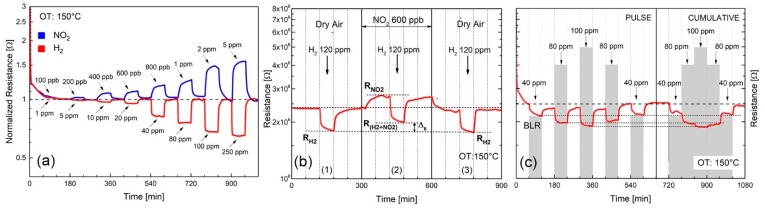
Electrical responses of the exfoliated WS_2_ post-annealed at 180 °C, at 150 °C operating temperature in dry air. (**a**) Comparison of the normalized dynamic response to NO_2_ (100 ppb–5 ppm) and H_2_ (1–250 ppm), (**b**) NO_2_ cross-sensitivity to H_2_: first panel, the response to 120 ppm H_2_ in dry air, second panel, response to 120 ppm H_2_ with 600 ppb NO_2_, third panel, response to 120 ppm H_2_ (as to first panel) for comparison, (**c**) Reproducibility and baseline recovery by exposing the film to both pulse and cumulative H_2_ concentrations in the range 40–100 ppm. H_2_ concentrations are highlighted in the figure by grey shadowed rectangular plots.

Figure 8a shows the normalized dynamic resistance changes, at 150 °C OT of few-layers WS_2_ thin films to NO_2_ and H_2_ in the 100 ppb–5 ppm and 1 ppm–250 ppm gas concentration ranges, respectively. WS_2_ films respond as *n*-type semiconductors with decreasing/increasing resistance upon exposure to H_2_/NO_2_, respectively. Degassing with dry air at 150 °C OT, the baseline resistance (BLR), as indicated by the black dotted line in the figure, is almost recovered. WS_2_ flakes are more sensitive to NO_2_ than H_2_ gas, with associated low detection limits (LDL) of 200 ppb and 5 ppm respectively, confirming what has been previously reported in the literature.

Cross-sensitivity, which represent the ability of WS_2_ to detect H_2_ in the presence of NO_2_ interfering gas, has been shown in Figure 8b. Panel (1) of Figure 8b shows the WS_2_ response to H_2_ alone in dry air carrier at 150 °C OT. The “cross-sensitivity” produced by interfering NO_2_ to the measure of H_2_ is displayed in panel (2) of Figure 8b. By exposing to 600 ppb NO_2_, sensor resistance initially increases, yielding at equilibrium the resistance value R_NO2_. As soon as 120 ppm H_2_ is introduced, the resistance decreases, yielding the equilibrium value shown as R_(H2+NO2)_. The cross-sensitivity effect is displayed in the picture as (ΔR), to indicate the gap between the electrical resistance to 120 ppm H_2_ alone (i.e., R_H2_) and that in the presence of 120 ppm H_2_ and 600 ppb NO_2_ (i.e., R_(H2+NO2)_). These results imply that the response to 120 ppm H_2_ is affected by the presence of a small amount (i.e., 600 ppb), of NO_2_ interfering gas, confirming the stronger affinity of WS_2_ to detect NO_2_ as respect to H_2_.

Reproducibility of the electrical response to pulse (on/off) and cumulative modes H_2_ gas adsorption is shown in Figure 8c, demonstrating acceptable response characteristics to H_2_. Under pulsed conditions, the baseline resistance (BLR) fully regains its initial value after completion of each desorption cycle in dry air. Under cumulative stepwise adsorption/desorption mode, the H_2_ gas resistance increases/decreases steadily, matching almost the same H_2_ resistance values obtained under pulsed conditions (black lines at saturation correspond to 40, 80 and 100 ppm H_2_).

Selectivity tests to both oxidizing and reducing gases carried out at 150 °C operating temperature with respect to 5 ppm NO_2_, exhibit satisfactory WS_2_ selectivity to both 5 ppm H_2_ and NH_3_ gases and to 250 ppm ethanol and acetone, as shown in Supplementary Figure S6. These data were demonstrated to be in line with previous research on WS_2_ nanoflakes synthesized by electrospinning [41].

**Figure 9:**
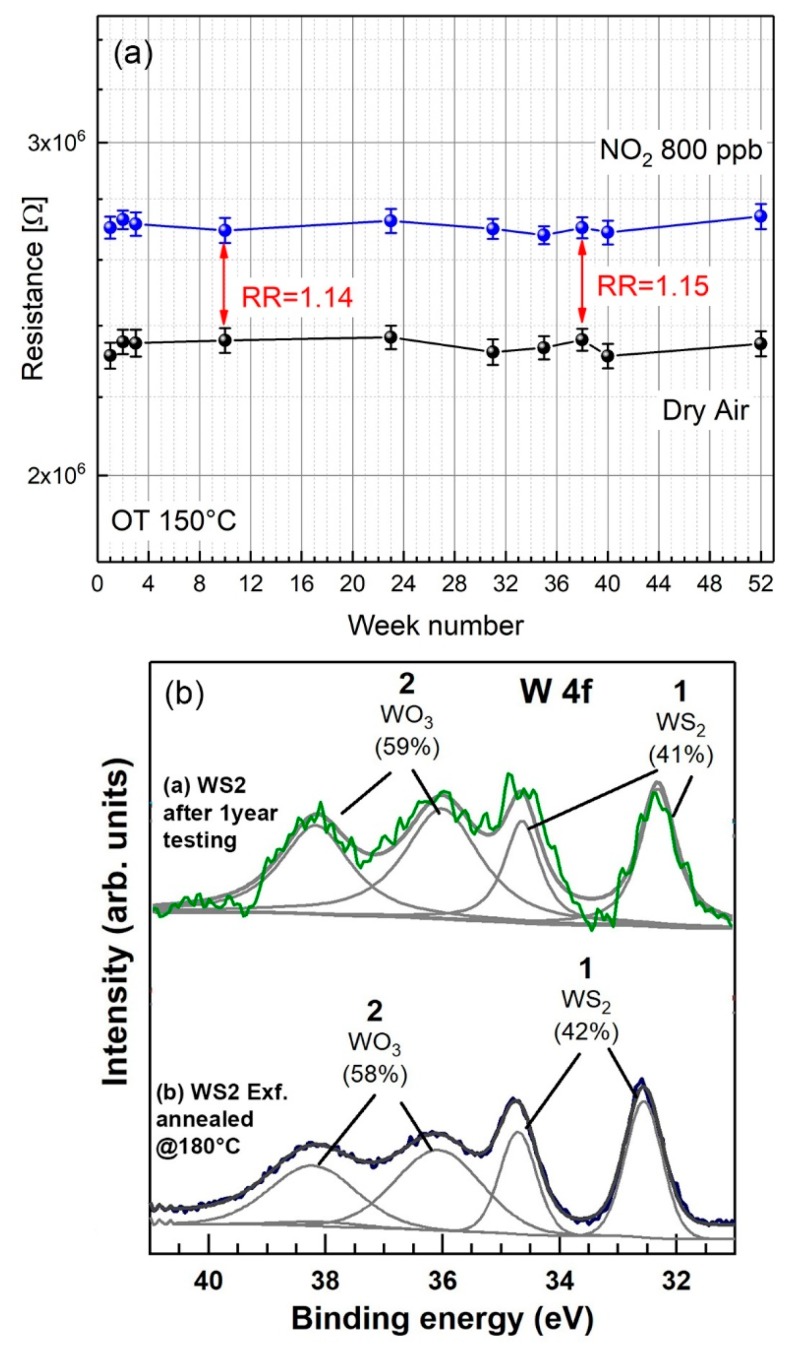
WS_2_ exfoliated and post-annealed at 180 °C. (**a**) Long-term stability properties of the electrical resistances of the baseline (lower curve) and 800 ppb NO_2_ over a period of 12 months (equivalent to approximately 5 months of continuous operation at 150 °C operating temperature). Average resistance values with associated standard deviations are calculated over a set of 5 consecutive measurements. (**b**) Comparison of the XPS signals of the as-exfoliated WS_2_ annealed at 180 °C (lower curve) and the same sample after one-year conditioning to various gases and 150 °C operating temperature.

Long-term stability properties of the electrical response of both baseline and saturation resistances to 800 ppb NO_2_ over a period of 12 months (corresponding to approximately 5 months of cumulative operations at 150 °C operating temperature) were also recorded. Figure 9a shows baseline resistances (lower curve) and saturation resistances corresponding to 800 ppb NO_2_ (upper curve), randomly collected over a period of 52 weeks. Average resistances with associated standard deviations are calculated over a set of 5 consecutive measurements. Relative responses (RR) taken over the investigated period are also highlighted in the figure. No remarkable fluctuations of both baseline and resistances at saturation were detectable, attesting good long-term stability of the electrical properties of the WS_2_ films. To validate the electrical responses shown in Figure 8a, we also investigated the oxidation state of the sensor surface, measuring the WO_3_ content before and after 52 weeks conditioning. Figure 9b compares the XPS W 4f signal of the as-exfoliated WS_2_ 180 °C annealed film before (lower curve) and after 52 weeks long-term conditioning to different gases and 150 °C OT (upper curve). Beside an increase of the signal noise after long-term conditionings, no substantial increase of the WO_3_ content was detected. These observations imply that exfoliated WS_2_ films, previously stabilized at 180 °C, can satisfactorily respond to different gases under sustained conditions at 150 °C operating temperature.

**Figure 10:**
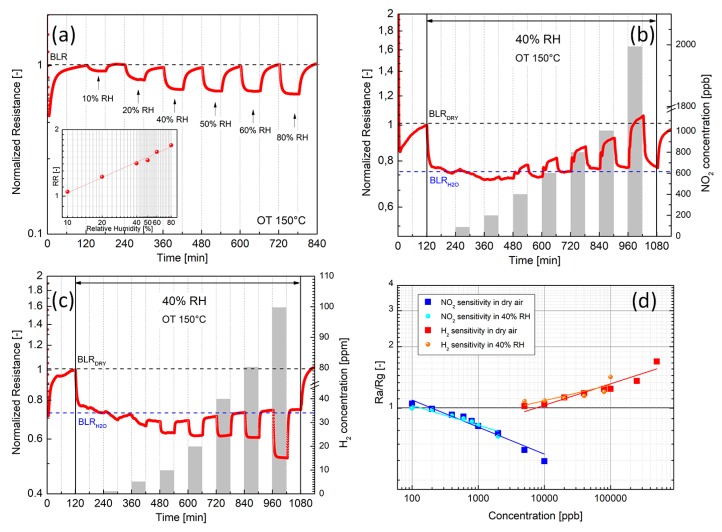
Electrical responses of the exfoliated WS_2_ post-annealed at 180 °C (150 °C operating temperature) to different Relative Humidity (RH) conditions. (**a**) Normalized dynamic response to humidity (10–80% RH). The inset depicts the corresponding sensitivity plot. (**b**) Dynamic response to increasing NO_2_ concentrations in air with 40% RH, (**c**) Dynamic response to increasing H_2_ concentrations in in air with 40% RH, (**d**) Comparison of the sensitivity plots to NO_2_ and H_2_ in dry air and 40% RH, respectively.

Finally, the influence of humidity on NO_2_ and H_2_ gas response at 150 °C OT is also reported. Figure 10 shows the dynamic response of the films exposed to air with increasing amounts of humidity, in the 10–80% relative humidity (RH) range. The inset of Figure 10a displays the related sensitivity plot. Considering that at 150 °C operating temperature physisorbed water is reasonably evaporated, it is unlikely that the decrease of the resistance is induced by a protonic surface charge-transfer mechanism, as reported for WS_2_ humidity sensors operating at room temperature [42]. More reasonably, water vapor at 150 °C OT behaves like a reducing gas inducing a steady resistance decrease in WS_2_ *n*-type semiconductor, which does not saturate with increasing the RH, as shown in the inset of Figure 10a.

In order to evaluate the influence of humidity to NO_2_ and H_2_ gases response, cross-sensitivity tests have been performed. Figure 10b, c shows the dynamic responses to different NO_2_ and H_2_ concentrations, measured in humid air at 40% RH. As soon as water vapor is introduced, a downshift of the baseline from BLR_DRY_ to BLR_H2O_ is shown. NO_2_ and H_2_ dynamic responses in dry and 40% RH are almost similar, as shown in Figure 8a and Figure 10b,c. Comparison of the NO_2_ and H_2_ gases sensitivities in dry and 40% RH air are shown in Figure 10d. Notably, no significant differences are displayed as respect to slopes of the calibrating curves (i.e., sensitivity) and relative responses values (i.e., Ra/Rg), attesting that both NO_2_ and H_2_ measurements are not affected by the presence of moisture.

These results, indeed, demonstrate the possibility to produce efficient and reproducible gas sensors, able to detect NO_2_ and H_2_ with no significant cross-sensitivity effects induced by humid air in the 10–80% RH range and 150 °C operating temperature.

### 3.4. Gas Response Mechanism

As previously discussed, by annealing the exfoliated WS_2_ at 180 °C a “surface layer” containing ≈ 58% of amorphous WO_3_, penetrating at last 10 nm from the surface, is formed. It turns out that both structure and chemical composition of the “Surface layer”, comprising crystalline WS_2_ and amorphous WO_3_, strongly influence the gas response mechanism.

**Figure 11:**
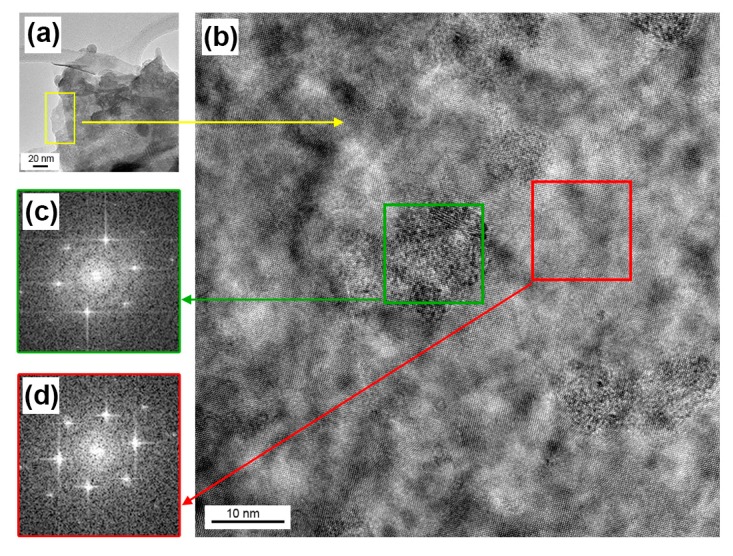
HRTEM images of the WS_2_ film pre-annealed at 180 °C. (**b**) Magnification of the yellow area of Figure 11 (**a**) displaying the presence of ordered structures (i.e., inside the red square) referred to crystalline WS_2_ and the presence of disordered ones (i.e., inside the green square) attributed to amorphous WO_3_. Related Selected Area Electron Diffraction (SAED) patterns are shown in (**c**) and (**d**), highlighting the occurrence of sharper reflections (**d**) associated to crystalline WS_2_.

HRTEM investigations of the pre-annealed 180 °C sample shown in Figure 11, display the occurrence of a complex surface patchwork comprising amorphous WO_3_ regions (located inside the green square of Figure 11b), possibly rearranging as not-connected, amorphous, isolated-clusters which are eventually embedded in crystalline WS_2_ phase (located inside the red square of Figure 11b).

To investigate the contribution of single WO_3_ on to the electrical properties of WS_2_/WO_3_ pre-annealed composite, we have prepared a fully-oxidized WS_2_, containing ≈ 99% amorphous WO_3_ and tested to NO_2_. This sample has been prepared by the same exfoliation method by grinding and drying WS_2_ powders at 25 °C, but setting the sonicating temperature at 60 °C, instead of 25 °C for 90 min, and finally pre-annealing in dry air at 180 °C.

**Figure 12:**
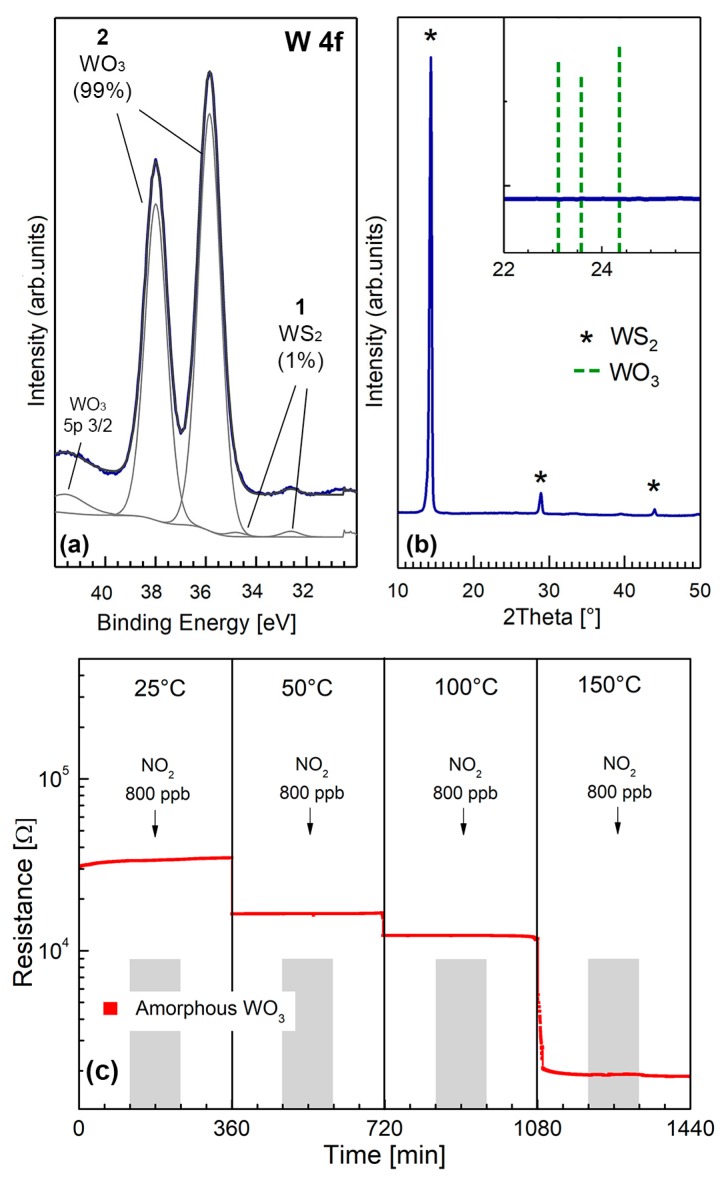
Chemical composition, crystalline structure and microstructural features of a fully oxidized WO_3_ thin film. (**a**) W 4f core level XPS spectra, (**b**) XRD grazing incidence spectra. Top right inset shows the close up of the 2θ region characteristic of crystalline WO_3_ (corresponding peaks of crystalline WO_3_, according to ICDS 98-001-7003, are highlighted by dashed green lines), (**c**) electrical response of the fully oxidized WO_3_ amorphous film to NO_2_ and different OTs.

Figure 12a,b shows the XPS and grazing incidence XRD patterns of the fully oxidized film, attesting that the chemical composition of the “surface layer“ is ≈ 99% WO_3_ (Figure 12a) and that the as-formed WO_3_ is amorphous, as highlighted by the absence of WO_3_ peaks inside the inset of the XRD pattern of Figure 12b. It turns out that the fully oxidized region possibly covers the whole surface of the flakes, not extending to the core, which maintain the crystalline structure of pristine exfoliated WS_2_ (as attested by the presence of WS_2_ peaks in the XRD pattern of Figure 12b). The electrical response of the fully oxidized amorphous WO_3_ to 800 ppb NO_2_ and different operating temperatures in dry air is shown in Figure 12c. According to Figure 12c, amorphous WO_3_ is not responsive to NO_2_ gas in dry air, in the operating temperature range 75–150 °C.

Literature reports discussing the gas sensing properties of amorphous WO_3_ are very scarce. Some authors found no gas response to NO_x_ of amorphous WO_3_ deposited by sputtering [43] whereas others demonstrated negligible NO_2_ response using photochemically-produced amorphous WO_3_ [44]. In most cases, the NO_2_ gas response of amorphous WO_3_ is smaller with respect to crystalline WO_3_, frequently associated with baseline drift phenomena, with few exceptions, mostly related to the preparation conditions. In our case, we demonstrated that the interaction of NO_2_ with amorphous 99% WO_3_ has no effects altogether.

Having shown in Figure 8a the substantial NO_2_ and H_2_ gas response of the WO_3_/WS_2_ composite, we conclude that it is crystalline WS_2_ which primarily respond to NO_2_ gas. The predominant gas sensing role played by crystalline WS_2_ with respect to amorphous WO_3_, is also supported by the decrease of the electrical resistance with increasing relative Humidity (RH), as shown in Figure 10a. Considering that WO_3_ interacts with humidity by increasing the resistance, due to WO_3_ lattice oxidation induced by humidity, as reported in the literature [45], the resistance decrease displayed in Figure 10a rules out any significative contribution of WO_3_ to the overall humidity response. Moreover, the hypothesis that WS_2_ is likely to be the responding material is also supported by our previous research, which demonstrated that humidity decreases the sensor resistance in MoS_2_-based exfoliated [25].

Discussing the contribution of crystalline WS_2_ to the overall electrical resistance, it was recently demonstrated by first-principles calculations on single MoS_2_ sulphur-defective layer that O_2_ irreversibly chemisorbs on sulphur vacancies [46] and that the “heal” of these defects by substitutional O atoms is thermodynamically favorable [47]. Furthermore, in case of direct NO_2_ molecules interaction with sulphur vacancies, a dissociative adsorption of NO_2_, leading to O atoms passivating the vacancies, and NO molecules physisorbed on the MoS_2_ surface, was also proposed [48,49]. Given these premises, we hypothesize that both O_2_ and NO_2_ suppress sulphur vacancies, supporting a gas response mechanism based only on physisorption of NO_2_ and H_2_ molecules on passivated (i.e., defect-free) WS_2_ surface. This hypothesis is sustained by theoretical studies on the adsorption of NO_2_, H_2_, O_2_, H_2_O, NH_3_ and CO gases on defect-free single layer MoS_2_ and WS_2_ [50,51]. According to this physisorption model, the size and sign of the resistance changes, when exposing few-flakes of WS_2_ to oxidizing (NO_2_) and reducing (H_2_) gases, depend on the number of exchanged carriers (i.e., electrons) and their direction. NO_2_ being more electronegative than H_2_ induces a large electron withdrawal, whereas H_2_ results in weak electron injection, explaining the increase/decrease of electrical resistance in *n*-type WS_2_, as well as the smaller detection limit measured for NO_2_ (200 ppb) as compared to the one found for H_2_ (i.e., 5 ppm).

Lastly, a question to be resolved is why both NO_2_ and H_2_ sensitivities are not affected by the presence of moisture, as shown in Figure 10d. This behavior may suggest that water vapor adsorbs on to the WS_2_ surface with a different and non-competitive mechanism with respect to NO_2_ and H_2_ gases. Clearly this interaction is a complex issue, and is yet to be clarified based on specific theoretical and experimental studies.

## 4. Conclusions

In conclusion, we have demonstrated an effective, reproducible and high-yield exfoliation process, obtained by enhanced low energy ball milling and sonication. Specifically, the two-step exfoliation followed by drop casting the centrifuged suspension leads to the deposition of thin films of well-packed and interconnected WS_2_ flakes with controlled and reproducible microstructure over large areas, thus representing a fast, simple and scalable method, compatible with standard microelectronic fabrication techniques. We found that a spontaneous oxidation of WS_2_ leading to the formation of amorphous WO_3_ on the surface of the exfoliated WS_2_ takes place, addressing the crucial drawback of surface oxidation of TMDs. We also found that by pre-annealing the WS_2_ films at 180 °C, a reproducible surface oxidation of WS_2_ to amorphous WO_3_ takes place, which stabilize from further oxidation the WS_2_ layers. Reproducible gas sensing responses to NO_2_ and H_2_ and humidity at 150 °C operating temperature were achieved with detection limits of 200 ppb and 5 ppm to NO_2_ and H_2_, respectively. The cross-sensitivity test highlighted a weak interference played by NO_2_ to the H_2_ gas response. Water vapor at 40% RH also resulted in having no interference to the measure of NO_2_ and H_2_ gases, attesting promising characteristics of WS_2_ exfoliated films for gas sensing applications.

## Supplementary Materials

The following are available online at https://www.mdpi.com/2079-4991/9/10/1363/s1, Figure S1: Schematic illustration of the exfoliation process, Figure S2: Schematic illustration of the gas sensing equipment, Figure S3: XPS spectra of S 2p core level, Figure S4: Grazing incidence XRD spectra of the as-exfoliated WS_2_; as-exfoliated WS_2_—200 °C annealed for 1 h, Figure S5: The electrical response of WS_2_ post-annealed at 180 °C at different operating temperatures and 800 ppb NO_2_ in dry air, Figure S6: Selectivity response of WS_2_ post-annealed at 180 °C at 150 °C operating temperature.
