# Supplementary material for: Two-Step Exfoliation of WS2 for NO2, H2 and Humidity Sensing Applications
Source: Nanomaterials (Basel). 2019 Sep 24;9(10):1363. doi: 10.3390/nano9101363 (PMC6835748; doi:10.3390/nano9101363)
Supplement: Supplementary file 1 [file nanomaterials-09-01363-s001.pdf]

**SUPPORTING INFORMATION**

# Two-step exfoliation of $WS_2$ for $NO_2$, $H_2$ and humidity sensing applications

**Valentina Paolucci [1], Seyed Mahmoud Emamjomeh [1], Michele Nardone [2], Luca Ottaviano [2,3] and Carlo Cantalini [1,*]**

[1] Department of Industrial and Information Engineering and Economics, Via G. Gronchi 18, University of L'Aquila, I-67100 L'Aquila, Italy; e-mail@e-mail.com

[2] Department of Physical and Chemical Sciences, Via Vetoio 10, University of L'Aquila, I-67100 L'Aquila, Italy; e-mail@e-mail.com

[3] CNR-SPIN Uos L'Aquila, Via Vetoio 10 I-67100 L'Aquila, Italy; e-mail@e-mail.com

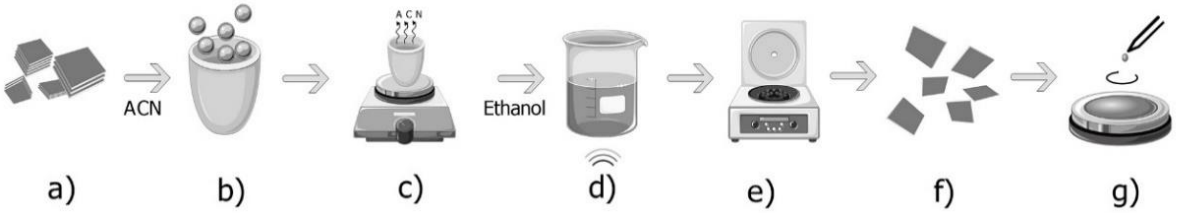


**Supporting Figure S1.** Schematic illustration of the exfoliation process. 2 g of WS2 commercial powder with 99% purity and average particle size of 2μm (a) were dispersed in 4 ml of Acetonitrile and ball milled (b) in a planetary milling machine at 400 rpm with with 30g Zirconium Oxide balls (D=3 mm) for 2 hours (steps of 15 min milling and 5 min idle to avoid excessive warming). The ACN residuals were then evaporated overnight (i.e. drying) at room temperature (c). After ACN evaporation, 0.05 g of the ball milled powder dispersed in 100 ml of pure ethanol and probe sonicated (d) for 90 minutes in a cool bath (T 25°C). Finally, the sonicated dispersion was centrifuged at 2500 rpm for 40 min at 20°C (e) and the supernatant collected (f) and spin coated on a proper substrate (g). Some drawings of this figure are distributed by "Servier Medical Art by Servier".

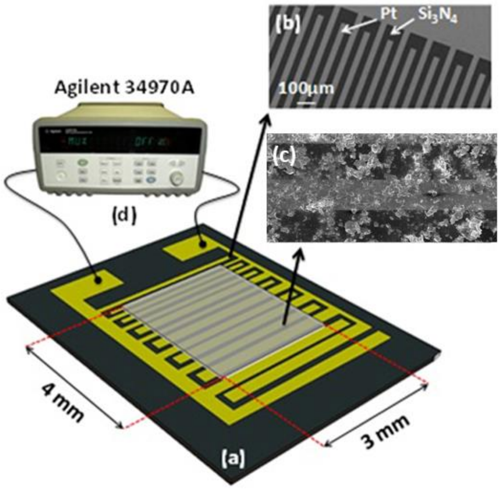


**Supporting Figure S2.** Schematic illustration of the substrate with drop cast exfoliated WS2: (a) Substrate front side showing the deposited area (light grey); (b) SEM picture of the Pt finger-type electrodes on Si3N4 substrate; (c) SEM picture of the 180°C annealed exfoliated WS2; (d) electrical connections to measure the resistance of the WS2 film by volt-amperometric technique.

The electrical resistance of the films was measured by an automated system. Thin film deposited on $Si_3N_4$ provided with Pt finger type electrodes was placed inside a Teflon chamber (500 $cm^3$), provided with Teflon tubings and exposed to gaseous mixtures containing between 1 ppm to 500 ppm $H_2$, between 40 ppb to 10 ppm $NO_2$ in dry air carrier and air with relative humidity in the range 10-80% Relative Humidity (RH) at 500sccm/min flow rate. The average gas residence time inside the chamber is set at 1 min. Different gas concentrations were obtained by mixing certified $H_2$ and $NO_2$ mixtures (500 ppm $H_2$, and 10 ppm $NO_2$ in dry air cylinder) with dry air carrier, by means of an MKS147 multi gas mass controller. Thin film electrical resistance was measured by means of the volt-amperometric technique (AGILENT 34970A) at different OTs in the temperature range 25°C-150°C by heating the $Si_3N_4$ substrate through back-side-circuit dc current injections (20–80 mA) as shown in Figure S2. The time scale for gas adsorption and air desorption was fixed at 1 h. Gas response properties have been discussed considering the following features: Base Line Resistance (BLR), i.e. the resistance in dry air at equilibrium; Relative Response (RR), i.e. the ratio ($R_G/R_A$) or ($R_A/R_G$) for a given concentration of oxidizing or reducing gases respectively.

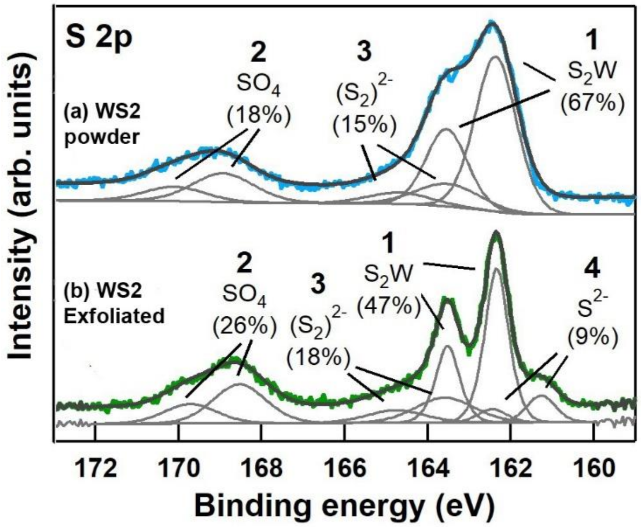


**Supporting Figure S3.** XPS spectra of S 2p core level acquired respectively on (a) pristine $WS_2$ commercial powder ($WS_2$ PWD); (b) $WS_2$ exfoliated by ball milling, drying and sonication at 25 °C

The XPS analysis of the S 2p core level spectra reported in Figure S3 shows the presence of several chemically shifted components, composed of the $2p_{3/2}$ and $2p_{1/2}$ doublets. These components are very likely a consequence of the high reactivity and oxidation states number of sulfur. The spectra are characterized by three main components, assigned to $WS_2$ (component 1, at 162.3 eV), to $SO_4$ (component 2, at 168.8 eV) and to non-metallic disulfide compounds $(S_2)^{2-}$ (component 3, at 163.7 eV). The presence of $SO_4$ and $(S_2)^{2-}$ on the surface of the pristine powder is due to air oxidation processes. All the assignments of the S 2p components are in line with literature.[1,2]

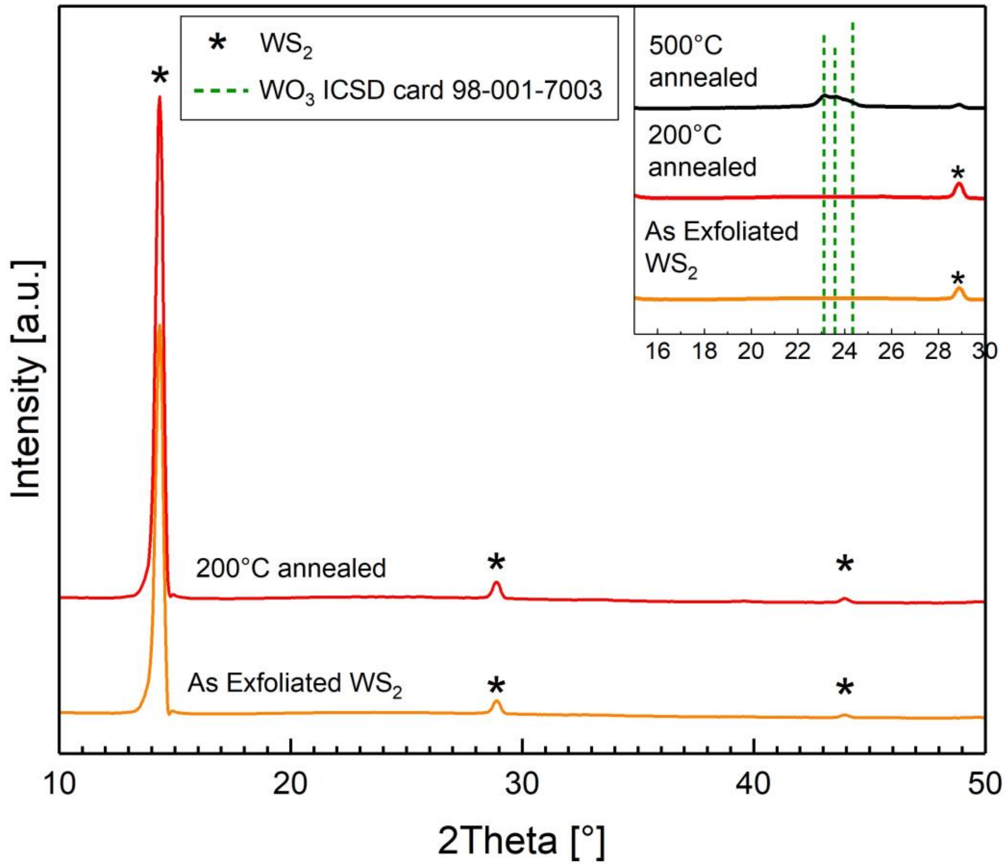


**Supporting Figure S4.** Grazing incidence XRD spectra of the as-exfoliated $WS_2$; as-exfoliated $WS_2$ - 200°C annealed for 1 hour. Top right inset shows the close up of the 2θ region characteristic of crystalline $WO_3$. Peaks of crystalline $WO_3$, according to ICDS 98-001-7003, are highlighted by dashed green lines. Notably that after annealing at 500 °C for 1 hour, $WO_3$ crystalline is formed, whereas the $WS_2$ peak located at 2θ degree 29° disappears.

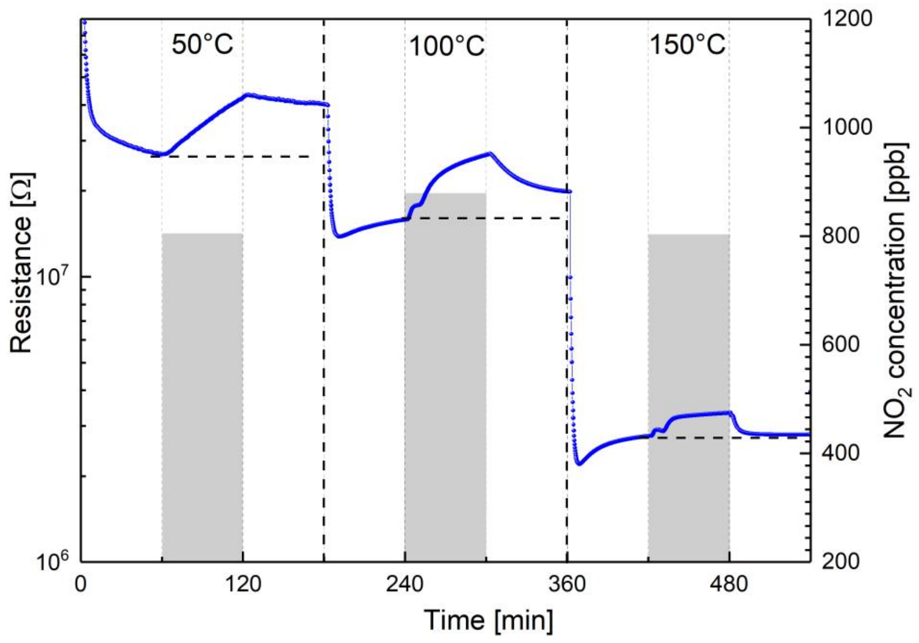


**Supporting Figure S5.** The electrical response of $WS_2$ post-annealed at 180 °C at different operating temperatures and 800 ppb $NO_2$ in dry air. Horizontal black dotted lines mark the base line resistances. $NO_2$ pulses are represented by the grey shaded rectangular plots. The time scale of the experiment consists of 1 hours conditioning in dry air and 1-hour exposure to $NO_2$ gases respectively.

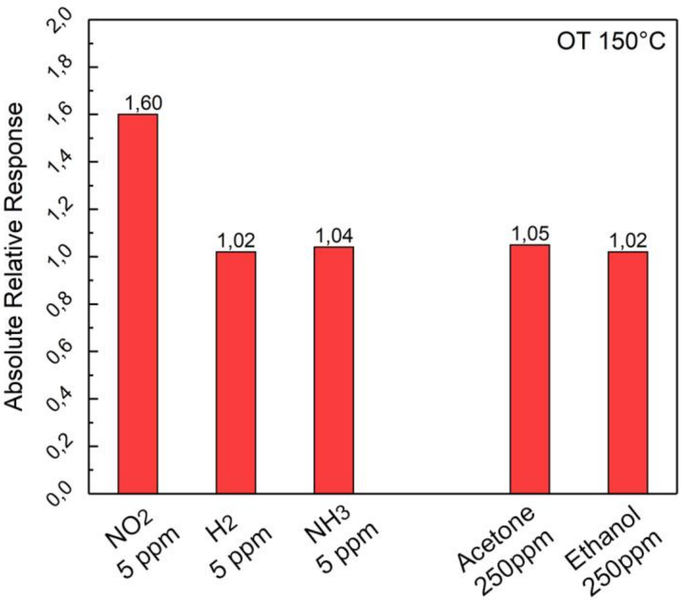


**Supporting Figure S6.** Selectivity response of $WS_2$ post-annealed at 180°C at 150°C operating temperature, respect to 5ppm $NO_2$. $WS_2$ film shows satisfactory selectivity to both 5 ppm $H_2$ and $NH_3$ gases and to 250 ppm ethanol and acetone.

# References

[1] Perrozzi F, Emamjomeh S M, Paolucci V, Taglieri G, Ottaviano L and Cantalini C 2017 Thermal stability of WS2 flakes and gas sensing properties of WS2/WO3 composite to H2, NH3 and NO2 *Sensors Actuators, B Chem.* **243**

[2] Benoist L, Gonbeau D, Pfister-Guillouzo G, Schmidt E, Meunier G and Levasseur A 1995 X-ray photoelectron spectroscopy characterization of amorphous molybdenum oxysulfide thin films *Thin Solid Films* **258** 110–4

[3] Jha R K and Guha P K 2016 Liquid exfoliated pristine WS $_2$ nanosheets for ultrasensitive and highly stable chemiresistive humidity sensors *Nanotechnology* **27** 475503
